# Predicting long-term pain by combining brain imaging, genetics and health questionnaire data with Swedish national registries using a prospective superstruct design

**DOI:** 10.1177/17448069241301628

**Published:** 2024-11-29

**Authors:** Filip Gedin, Sebastian Blomé, Granit Kastrati, Maria Lalouni, Fredrik Åhs, Peter Fransson, Jörgen Rosén, William Hedley Thompson, Karin Jensen

**Affiliations:** 1 Department of Clinical Neuroscience, Karolinska Institutet, Stockhom, Sweden; 2 Department of Psychology and Social Work, Mid University Sweden, Östersund, Sweden; 3 Department of Applied Information Technology, Gothenburg University, Gothenburg, Sweden

**Keywords:** Pain, neuroimaging, functional magnetic resonance imaging, risk factors, demography

## Abstract

**Background:**

Long-term pain is a common health problem that results in disability for patients of all ages, leading to an enormous economic burden. Over 20% of the population suffer from long-term pain. Unfortunately, there are no clinical tests that predicts who will develop long-term pain. The overall aim is to predict future pain incidence based on brain function, pain behavior, health status, and genetic variability.

**Method:**

PrePain utilizes a superstruct design, which involves recruiting participants from ongoing research projects. Eligible individuals for participation in PrePain were over 18 years old and free from long-term pain. During the baseline visit, participants provide pain and health-related questionnaires, undergo structural and functional MRI scans, and provide a saliva sample for DNA extraction. Individual baseline measures are then routinely followed-up via national registries.

**Result:**

We present quality-assessed data from over 300 participants. The average age was 34 years, and most participants were women (75%). Participants rated their pain sensitivity above average and reported low avoidance. Catastrophizing thoughts during painful episodes were rated as moderate. Assessments of (f)MRI data indicated generally good image quality. In this first follow-up, we found that 45 participants had a pain-related diagnoses.

**Conclusion:**

Results indicate that a superstruct design is feasible for collecting a large number of high-quality data. The incidence of long-term pain indicates that a sufficient number of participants have been recruited to complete the prediction analyses. PrePain is a unique prospective pain database with a fair prognosis to determine risk factors of long-term pain.

## Introduction

Long-term pain is a common health problem that causes disability in patients, leading to an enormous economic burden, and loss of productivity in working-age groups.^1^ At any given timepoint, more than 20% of the population suffer from a long-term pain problem.^2^ In order to avoid chronicity, identifying those with high risk is essential to provide preventive care and decrease pain incidence.^3^ There are no clinical tests that predict who is going to develop long-term pain, although previous attempts have been made to predict future severity of pain in already affected patients.^4,5^

Epidemiological studies have provided important information about associations between pain and sociodemographic factors^6–8^ and psychiatric comorbidity.^9–11^ Generally, gender and age are factors associated with long-term pain.^12,13^ However, association studies are not prospective, as they cannot determine causality, and the suggested risk factors are not precise enough to develop tools that screen for pain vulnerability. Prospective data for the development of long-term pain are scarce. In a large community-based survey, lifestyle and psychosocial risk factors were emphasized as a contribution to long-term pain.^14^

Cross-sectional comparisons of the brain’s anatomy and physiology between patients with long-term pain and controls have found markers of pain pathology, including altered brain activations,^15^ brain volumes^16^ and different levels of inflammatory agents in the central nervous system.^17^ Yet, prospective risk factors are unknown. Attempts to predict long-term pain has used experimental tests that tap into the neuronal signature of pain directly in both adults^18^ and in adolescents with sub-acute pain.^19^ However, the major limitation of these studies was that participants were patients who already had sub-acute pain, or were at high risk of pain, and that the sample sizes were small. To our knowledge, only one study has investigated the neural correlates to pain chronification in patients with sub-acute pain,^20^ where patients with persistent pain had higher brain activations in limbic brain circuitry compared to those in remission after 1 year.

In order to increase the statistical power and sensitivity to predict future long-term pain, *PrePain* uses a superstructure design, referring to a data collection that is piggybacking on an ongoing project’s data collection. Repurposing brain imaging data from other research projects at MRI facilities allows for a large sample to make a prospective neuroimaging study feasible. Combined with saliva samples and follow-ups in national health registries, *PrePain* creates an opportunity to provide predictors of long-term pain and address the limitations of conventional epidemiological and neuroimaging studies, respectively. This inherent connection between pain, cognition and emotion emphasizes the need for closer description of pain and frequency of psychiatric conditions.

The overall aim of *PrePain* database is to predict future pain incidence through large-scale exploration of the link between sociodemographic risk factors, pain behavior, and brain function, and in the future, genetic variation. In this study, the aim was to provide a comprehensive description and evaluation of data from the 300 first participants in the *PrePain* database.

## Methods

### Study design

**Figure 1.**
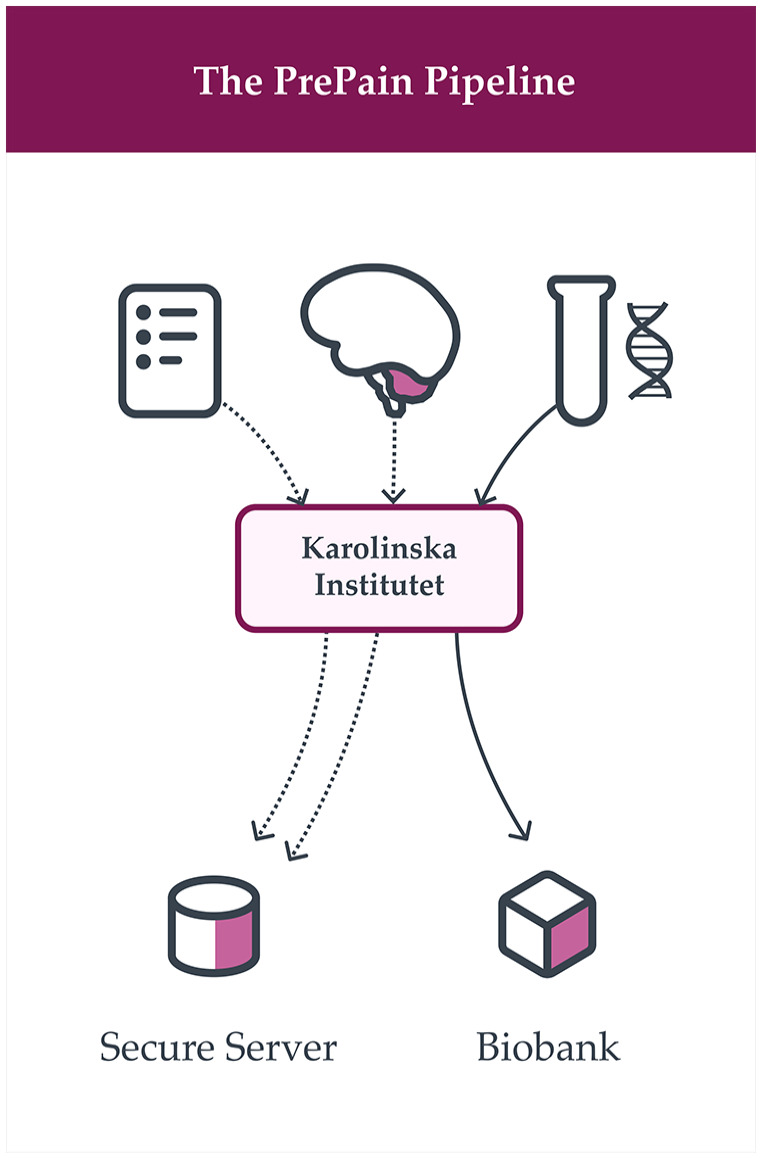
Schematic overview of the different data types included in *PrePain database*. At baseline, brain imaging data (structural and functional), surveys on pain-specific and general health-data, as well as saliva samples for future genetic analyses is collected from healthy individuals with no previous or ongoing pain problems. The saliva is banked at the Karolinska Institute biobank for future genotyping and the brain and survey data are banked at a secure server at the same institution. After the baseline visit, each participant will be followed-up via Swedish health registries to obtain information about long-term pain incidence or related diagnoses.

*PrePain* includes one visit at baseline. Repeated follow-ups are performed via regular queries in national health registries during the years to follow. At the baseline visit participants are asked to: (1) give informed consent, (2) complete pain and health-related surveys (*PrePain* questionnaire), (3) undergo structural and functional MRI scans, (4) provide a saliva sample for future genetic analyses (this process is illustrated in Figure 1).

A superstruct design is used to collect *PrePain* data at baseline, meaning that a large number of structural and resting-state functional MRI images will be collected from healthy participants that take part in other research studies at brain imaging facilities in the Stockholm area. In some studies, there is already a structural and functional MRI scan included in the participating project, and in other studies these images are added for the *PrePain* purpose.

Ethical approval was received from the Regional Ethical Review Board of Stockholm (#2018/2251-31/2) and the consent procedure includes a separate written and oral informed consent for *PrePain* even if a participant has already provided consent for the initial brain imaging study.

### Participants

Eligible participants must be over the age of 18 years and exclusion criteria include: having a long-term pain problem at the time of enrolment, prior history of neurological disorders, severe psychiatric disorders, or substance abuse. So far, >300 pain-free individuals have been recruited to *PrePain* with an increasing rate of recruitment since the end of the Covid-19 pandemic. Data collection is planned to continue consecutively until year 2029 with an estimated number of 3500 participants available for follow-ups in national health registries and, ultimately, genetic analyses. The renumeration for taking part in *PrePain* (independent from any remuneration from other studies) is given as two vouchers for cinema tickets (approximately 30 EUR).

### Brain imaging parameters

The *PrePain* brain image collection effort is built on a rapid acquisition protocol being added to existing neuroimaging research project of pain free individuals. The additional *PrePain* protocol includes a 3D T1-weighted structural image acquisition (6 min) and a resting-state functional MRI scan (8 min). This time effective imaging acquisition approach allows for very large number of individuals to be added to the PrePain database.

Brain imaging data is collected using two 3T scanners, a Discovery MR750 (GE Healthcare) and a MAGNETOM Prisma (Siemens Healthcare, Erlangen). The image acquisition protocol on each scanner site was harmonized to produce MR images of comparable quality (a detailed account of image acquisition parameters and protocol settings is given in Table 1).

**Table 1.** Scanner parameters for image acquisition at the two study sites.

Parameter	Karolinska Institutet MR-center Stockholm, Sweden	Stockholm University Brain Imaging Center Stockholm, Sweden
Status	Completed	Ongoing
Scanner	GE Healthcare Discovery MR750	SIEMENS MAGNETOM Prisma
Head coil	8-channel head coil	20-channel head coil
Functional scans		
TR	2205 ms	2210 ms
TE	30 ms	30 ms
Flip angle	80 degrees	80 degress
Slice thickness	3 mm	3 mm
Structural scans		
TR	8160 ms	2300 ms
TE	3180 ms	2960 ms
Flip angle	12 degrees	9 degrees
Slice thickness	1 mm	1 mm

Structural data include a high-resolution T1-weighted image. Functional imaging data were acquired using a gradient-echo echo-planar imaging (EPI) sequence sensitive to blood oxygenation level-dependent (BOLD) contrast. Images included coverage of the whole brain and the entire cerebellum. An 8-min resting-state BOLD scan was performed for each participant, resulting in 220 brain volumes. During BOLD data collection, participants were instructed to remain still, stay awake, and keep their eyes open, with their gaze directed to a crosshair, while blinking normally. Identical verbal instructions were used for the resting-state scans on at both sites.

### Preprocessing of neuroimaging data

The preprocessing of BOLD data is performed using fMRIPrep.^21^ fMRIPrep is a robust preprocessing pipeline for both task-based and resting-state fMRI. The fMRIPrep pipeline includes minimal preprocessing, including motion correction, field unwarping, spatial normalization, bias field correction, and brain extraction (for details, see Supplementary materials). fMRIPrep was chosen for its known robustness in providing comparable results independently of scanner manufacturer or scanning parameters. To assess the quality of the acquired images for the different sites, MRIQC is used to assess the quality of the images.^22^ To compare the quality of the *PrePain* dataset, we contrast the data for each site with the crowdsourced MRIQC dataset to identify subpar images.^21^

### Semi-automated preprocessing pipelines

**Figure 2.**
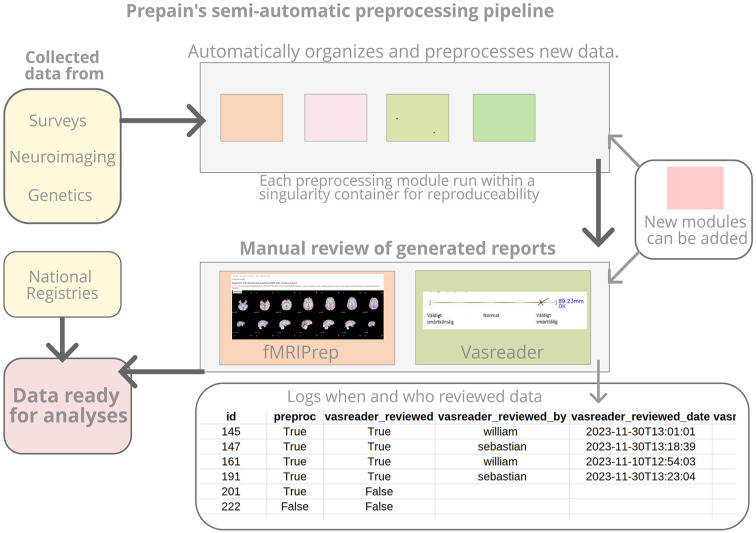
A flowchart describing semi-automatic preprocessing pipeline.

Ensuring data integrity across various scanners and data types is crucial for our project. Our modular, semi-automated preprocessing routine facilitates replicability and accommodates changes without compromising the overall database integrity. We use singularity containers to run each module, ensuring consistency of the preprocessing steps over time, as they might slightly change or new pipelines may be developed (each step is described in Figure 2).

Our semi-automated routine collects the data from the different sources (*PrePain* questionnaire data and fMRI data) preprocesses and reviews the data. For the latter, we use standard preprocessing pipelines such as *heudiconv* for transforming the dicom images into the Neuroimaging Informatics Technology Initiative file format (NIfTI), organized with the Brain Imaging Data Structure (BIDS), and fMRI Preprocessing (*fMRIPrep)*.^21^ For the questionnaire data, the Visual Analogue Scale Reader (*VASreader*) has been developed and is presented here for the first time. VASreader automatically decodes with precision estimates and produces a report for manual review. The semi-automated preprocessing pipelines and manual reviewing steps are regularly executed.

**Figure 3.**
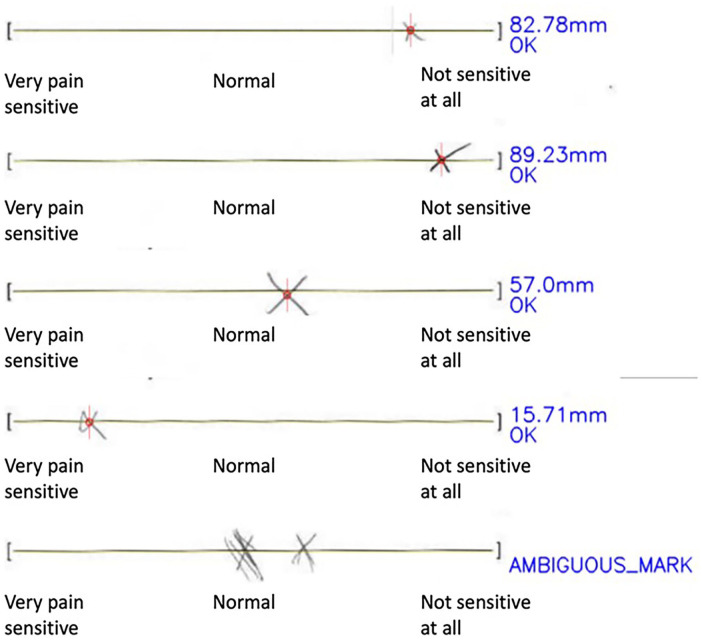
Several examples of *VASreader*’s generated reports for various types of input including marks that are small, skewed, offset from the line, intersect multiple times, and inputs that cannot be read. The preprocessing pipeline entails that each *VASreader* report should be reviewed and if any values do not seem accurate, need to get inputted manually. Any manual inputs are logged.

The *fMRIPrep* and *VASreader* reports are reviewed manually by our team upon completion and the reviewer can specify if the data is accepted, rejected, or flagged (Figure 3). The decision, the reviewer identity, and the date of the review all get logged. This helps ensure that all data has been reviewed.

This modular semi-automatic preprocessing system facilitates the addition of more modules, additional review steps, and other types of data (e.g. genetic data) without disrupting the rest of the pipeline. The setup also accommodates the addition of new sites as the *PrePain* superstruct project scales up.

### Self-rated quality of life and pain perception questionnaires

EQ-5D is an internationally standardized instrument developed to provide a generic measure of self-reported quality of life (http://www.euroqol.org). EQ-5D consists of five questions related to an individual’s health-related quality of life and a continuous visual scale for ratings of life. The EQ-5D questionnaire takes less than 5 min to complete and will be used to analyze inter-individual variability that might be predictive of long-term pain.

In order to verify that participants are truly pain-free at the time of inclusion in the *PrePain* database, we added an additional set of questions that is unique to the PrePain database. This set of questions is implemented as a short (less than 2 min to complete) questionnaire with questions related to different aspects of pain, such as current pain, pain history, heritability for long-term pain, pain sensitivity, fear of pain, etc. The questionnaire also includes questions about pain avoidance, pain catastrophizing, and health anxiety, as these are psychological factors previously associated with the transition from sub-acute to long-term pain.^23^ The *PrePain* questionnaire is currently being validated and the psychometric properties will be reported elsewhere.

### Saliva collection for DNA extraction and genotyping

Saliva is collected from each participant by using an Oragene saliva collection kit (www.dnagenotek.com). The kit consists of a small plastic tube in which the saliva can be collected via spitting. The saliva sample are currently being banked and will be used for genotyping and analyses of genetic contributions in the future.

### Registry data

Assessment at baseline will be combined with follow-up data from Swedish national registries. These registries provide a unique opportunity for retrieving individual health information without contacting participants after their baseline study visit, and therefore mitigate the risk of significant drop-out rate and missing data. The following registries are queried: From the *National Board of Health and Welfare*: The Drug register, Patient register, Cancer register, and Cause of death register. From Swedish Social Insurance Agency: The MicroData for Analysis of the Social Insurance database (MiDAS). From *Statistics Sweden*: The Longitudinal integrated database for health insurance and labour market studies (LISA) register. Taken together, these registries provide information about demography, socioeconomics, sickness absence, health care utilization, medical diagnoses, and mortality. Only data relevant to the *PrePain* research questions is extracted from the registries and the analyses are performed on anonymized data. Only the project PI has access to a code key that can link a person’s identity to any *PrePain* datapoint.

*PrePain* performs yearly automated follow-ups of its participants via regular queries from Swedish national registries. These follow-ups are essential for determine whether long-term pain, or psychiatric disorders, have developed since baseline. Lastly, the registries will provide information on how long-term pain impacts both health and cost (see Table 2).

**Table 2.** List of registries and type of information queried for follow-up data.

Government agency	Registry	Data
National Board of Health and Welfare	National Patient Registry	Inpatient and specialized outpatient healthcare
	Prescribed drugs register	Type of drug, quantity and dosage, reimbursement
	Cause of Death Register	Cause and date
Statistics Sweden	Longitudinal integrated database for health insurance and labour market studies (LISA)	Demographics and socioeconomic factors
Swedish Social Insurance Agency	National Social Insurance Agency Micro-Data for Analysis of the Social Insurance System (MiDAS)	Dates, main diagnosis, and days on sick leave

## Results

**Figure 4.**
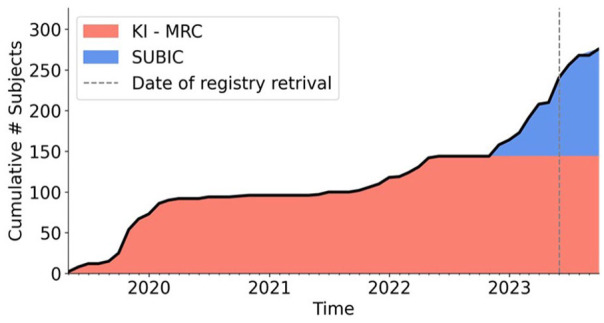
Number of participants included in the *PrePain* database for the different sites two scanning sites. Note the absence of new data being added to the database during the 2020–2021 time-period due to the COVID-19 pandemic. The dashed line indicates the date when data was queried to the national registries.

Since the data collection onset in 2019, notwithstanding the Covid-19 pandemic slowing down the data collection in 2020 and 2021, we have to date reached a total number of *n* = 300 participants (Figure 4).

As shown in Table 3, the proportion of women enrolled so far is 74.5%. The mean age of the enrolled participants was 34, with an age range of between of 17 and 74 years. The majority of participants reported having higher education and were gainfully employed. Most were born in Sweden (91%) and had residence in the Stockholm municipality (82%). Their self-reported health-related quality of life was 81 on an EQ-VAS scale, which is slightly higher than the general population of Sweden, which averages 76.6 (SD 18) for men and 75.7 (SD 19.0) for women.^24^

**Table 3.** Demographics for the *PrePain* dataset.

Age (SD)	34 (10)
Age range	19–74
% Female	74.5
% Education level	
Elementary school	7
High school	46
University/college	134
% Born in Sweden	91
% Living in Stockholm	82.4
% Gainfully employed	73
Health status EQ-VAS (SD)	81 (14.38)

### PrePain questionnaire results

**Figure 5.**
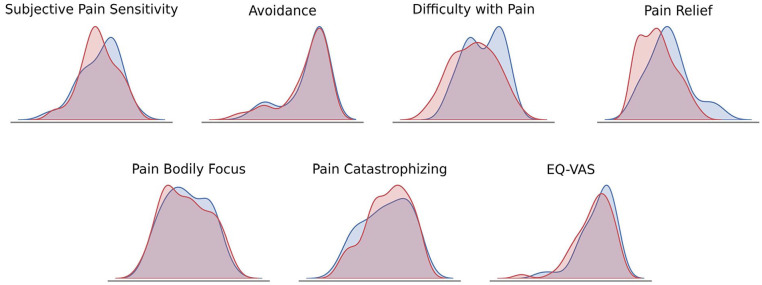
Density plots of *PrePain* questionnaire.

With regards to long-term pain, a majority of participants reported having no ongoing long-term pain (85%), although 15% responded that they had an ongoing long-term pain problem. At the time of the baseline assessments, 54% of the participants reported having no acute pain and 46% reported having some form of acute pain, for example, headache, tooth ache, or sprained joint. The distribution for the *PrePain questionnaire* results is presented in Figure 5.

The *PrePain questionnaire*, which includes pain-specific questions on pain-related attitudes and behaviors, revealed that, on average, participants rated themselves as being less sensitive to pain than others, (*M* = 61, SD = 20) on a 0–100 mm VAS ranging from 0 = very sensitive, to 100 = not sensitive at all (“How sensitive do you feel you are to pain compared to others”).

On average participants reported little avoidance of potentially painful situations (*M* = 84, SD = 20) on a 0–100 mm VAS ranging from 0 = always to 100 = never (“To what extent do you avoid situations where there is a risk of getting hurt [e.g. tattoos, football, visits to the dentist]”).

Regarding the aversiveness of pain, participants, on average, rated the experience of pain as moderately challenging, (*M* = 54, SD = 24) on a 0–100 mm VAS ranging from 0 = very hard to 100 = not hard at all (“How hard do you find it when you are in pain?”).

On average, participants reported feeling very relieved when pain subsides (*M* = 26, SD = 20) on a 0–100 mm VAS ranging from 0 = very relieved to 100 = not relieved at all (“How relieved do you feel when the pain goes away?”).

Regarding bodily focus during pain, participants reported a moderate level of bodily focus (*M* = 49, SD = 26) on a 0–100 mm VAS ranging from 0 = very much to 100 = not at all (“In general, how much do you focus on your body and its reactions when you think you are sick?”).

Participants reported having a moderate amount of catastrophizing thoughts during painful episodes (*M* = 65, SD = 24) on a 0–100 mm VAS ranging from 0 = always to 100 = never (“Does pain usually lead to negative thoughts that your health is getting worse?”).

### Quality of brain imaging data collected at baseline

**Figure 6.**
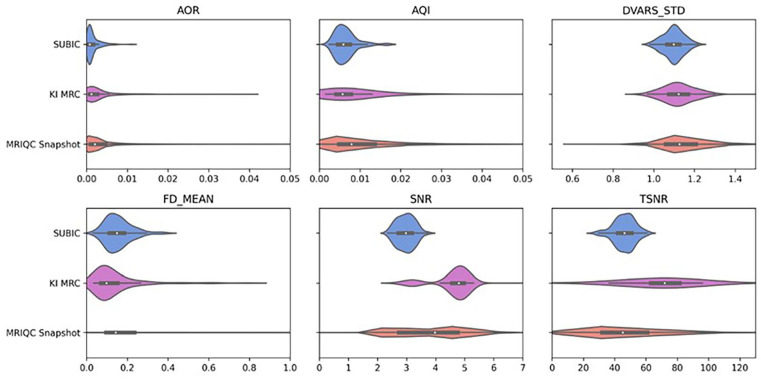
Brain imaging data quality. Violin plots depicting the distributions of various quality control metrics from MRIQC for the two sites currently in *PrePain* and a snapshot of crowdsourced MRIQC quality control metrics. Some outliers are truncated in order to show the majority of the distribution. AOR: AFNI’s outlier ratio; AQI: AFNI’s quality index; DVARS_STD: DVARS standard deviation of the temporal difference time series; FD_MEAN: framewise displacement mean; SNR: signal-to-noise ratio; TSNR: temporal SNR.

Figure 6 shows the distributions of quality metrics from MRIQC preprocessing pipeline for the two current scanning sites in *PrePain* database and contrasts our results with the crowdsourced snapshot of MRIQC users. The variables we selected to give an overview of the functional MRI data quality were: Afni outlier ratio (AOR), AFNI quality index (AQI), DVARS, Mean framewise displacement, derivative of root mean square variance over voxels (DVARS) normalized with the standard deviation of the temporal difference time series (DVARS_STD), the mean framewise displacement (FD_MEAN), the signal to noise ratio (SNR), and temporal signal to noise ratio (TSNR). See the MRIQC documentation for more information about each of these variables.

Overall, the results shown in Figure 6 suggests that the quality of the fMRI data in *PrePain* is good and comparable to that obtained from the crowdsourced snapshot of metrics. The degree of participant head-movement appears to be on par with crowdsourced data. However, we note that the signal-to-noise ration of the fMRI data scanned at the SUBIC scanning site is in the lower range, an observation that need to be kept in mind in future analyses of the data. Using a threshold of mean FD > 0.5, we have excluded data from four participants from further analysis.

### Follow- up results from registry-data – pain and psychiatric disorder incidence

From the start of *PrePain* inclusions, 11 participants were found to have received sick leave for a pain-related diagnosis, and 20 for mental health disorders. The average number of days individuals with pain were on sick leave was 67 days, ranging from 18 days to 343 days. For individuals with mental health disorders, the average number of days on sick leave was 156, with a range from 19 days to 803 days.

A total of 34 participants had received a pain-related diagnosis from inpatient or specialized outpatient healthcare after baseline.

## Discussion

### Feasibility

We have here provided evidence for the feasibility to collect prospective data for determining risk-factors for long-term pain. Truly prospective studies, where healthy individuals are studied from the time before developing a pain condition, are rare due to the large number of samples size required. This is particularly true for brain imaging data. Using a superstruct design, we were able to reach >300 participants at this initial follow-up. Quality controls revealed sufficient image quality despite the piggyback method where data was collected by staff employed in other research projects at two different scanning sites.

### Sufficient incidence of pain in our material

Determining the number of participants who developed a pain condition since baseline was a crucial point in of this first follow-up. To that end, registry data was available for 187 of the 300 enrolled participants. As expected, participants rated their health better than the Swedish average at baseline. Yet, we found a sufficient cumulative number of pain conditions, and mental health incidences, to reach sufficient statistical power in future predictive analyses. The enrolment began in 2019 and we conclude that the cumulative incidence will be sufficient for prediction analyses in 3–5 years, as we observe that approximately 5% of participants had been on sick leave for more than 14 days due to a pain related diagnose. The number of individuals with a pain related diagnose from specialized outpatient or inpatient healthcare was 34. This is in line with previous data on pain incidence in healthy individuals^20,25^ and provide an indication of the feasibility and statistical power in future predictive analyses. The registry data is crucial for understanding which participants have developed long-term pain and which socioeconomic factors that could impact its development.

### Are participants generalizable

The demographics of the first *PrePain* cohort reflect a largely well-educated group diverse in age. There is a gender imbalance that favors women which likely will even out as the project enrolls more participants and becomes increasingly more representative of the Swedish general population. Most of the participants had a university degree and were gainfully employed which suggests a higher economic status. Yet, 45% of swedes have a higher education and the vast majority are employed.^26^ There is a possibility of a regional bias as most participants live in Stockholm.

### Pain in the current cohort

The intention of *PrePain* is to enroll individuals without previous or ongoing long-term pain. Still, there was a small number of individuals who reported having long-term pain in our material. Furthermore, when asked specifically about any ongoing acute pain at baseline (e.g. temporary headache, tooth ache) almost half of the participants reported having some form of current pain. This could potentially be a limitation to our analyses as it may point toward an emerging subclinical pain problem already at baseline. Yet, the nature of the reported pain was very mild and the participants reported having a high health-related quality of life. The self-reports in the *PrePain* questionnaire, asking questions regarding pain sensitivity, avoidance behaviors, aversiveness, relief, bodily focus, and catastrophizing, indicated that participants rated less pain sensitivity, avoidance behaviors and catastrophizing compared to their estimation of pain in the general population. These psychological factors usually score higher in patients with long-term pain, and have also been suggested as predictors for long-term pain in individuals already suffering from acute or sub-acute pain.^27^

### Quality of MR-scans

Considering the superstruct design of the PrePain database, which included data from two MRI scanning sites and data collection conducted by staff members who were primarily employed to collect data for other studies, the overall imaging quality was good and the exclusion rate due to excessive head movement was small (four participants). The Signal to Noise ratio (SNR) for the imaging data from SUBIC were slightly lower than data from Karolinska Institutet, yet remains within acceptable limits. This should be controlled for in future analysis, even if it is unlikely to bias the prediction of risk for long-term pain.

### Study strengths and limitations

One of the limitations of the *PrePain* prospective design is the need for an inclusion of many participants. Since its inception in 2019, more than 300 participants have been enrolled in the database. This is well under our aim of including most of the 500 participants that every year undergo MRI scanning for research purposes at the Karolinska Institutet or SUBIC (Stockholm University). Notwithstanding the fact that data was not collected during the pandemic years and that it takes time to restart research projects that were temporally put on hold, the number of included participants in *PrePain* almost doubled during 2023, and we are predicting the same trend for the year 2024.

Major advantages of the *PrePain* database include its novel approach, leveraging a unique combination of advanced neuroimaging techniques, genotype data, registry-based data, and a large-scale collaboration between multiple brain imaging centers to investigate risk factors for long-term pain. Additionally, the study has the advantage of a substantial cohort size and access to comprehensive Swedish registry data, which provides a robust framework to examine both biological and socioeconomic factors that may influence pain onset. The outcome of our interdisciplinary approach is expected to significantly contribute to our understanding of long-term pain and provide pivotal insights into effective interventions aimed at preventing the development of long-term pain in individuals at risk. The increasing rate of participant recruitment, cumulative incidence of clinical pain, and the richness of available study data positions *PrePain* to make a meaningful impact on the field of pain research, offering hope for a future free from the debilitating consequences of long-term pain.

## Conclusion

In summary, we have shown that the prospective superstruct project *PrePain* is feasible and can provide valuable insight on determining risk-factors of long-term pain. The cohort is representative for the targeted population and the quality of imaging data collected from different centers are robust and of good quality.

## Supplemental Material

sj-docx-1-mpx-10.1177_17448069241301628 – Supplemental material for Predicting long-term pain by combining brain imaging, genetics and health questionnaire data with Swedish national registries using a prospective superstruct design Supplemental material, sj-docx-1-mpx-10.1177_17448069241301628 for Predicting long-term pain by combining brain imaging, genetics and health questionnaire data with Swedish national registries using a prospective superstruct design by Filip Gedin, Sebastian Blomé, Granit Kastrati, Maria Lalouni, Fredrik Åhs, Peter Fransson, Jörgen Rosén, William Hedley Thompson and Karin Jensen in Molecular Pain
